# Multi-Nutrient Fortified Dairy-Based Drink Reduces Anaemia without Observed Adverse Effects on Gut Microbiota in Anaemic Malnourished Nigerian Toddlers: A Randomised Dose–Response Study

**DOI:** 10.3390/nu13051566

**Published:** 2021-05-06

**Authors:** Adedotun J. Owolabi, Idowu O. Senbanjo, Kazeem A. Oshikoya, Jos Boekhorst, Robyn T. Eijlander, Guus A. M. Kortman, Jeske H. J. Hageman, Folake Samuel, Alida Melse-Boonstra, Anne Schaafsma

**Affiliations:** 1 FrieslandCampina WAMCO Nigeria Plc, Industrial Estate, Plot 7b Acme Rd, Ogba, Ikeja, Lagos 100001, Nigeria; adedotun.owolabi@wur.nl; 2 Division of Human Nutrition and Health, Wageningen University and Research, P.O. Box 17, 6700 AA Wageningen, The Netherlands; alida.melse@wur.nl; 3 Department of Paediatrics and Child Health, Paediatric Gastroenterology, Hepatology and Nutrition Unit, Lagos State University College of Medicine, Ikeja, Lagos 100001, Nigeria; senbanjo001@yahoo.com; 4 Department of Pharmacology, Therapeutic and Toxicology, Lagos State University College of Medicine, Ikeja, Lagos 100001, Nigeria; kazeemoshikoya@ymail.com; 5 NIZO Food Research B.V., 6718 ZB Ede, The Netherlands; Jos.Boekhorst@nizo.com (J.B.); Robyn.Eijlander@nizo.com (R.T.E.); Guus.Kortman@nizo.com (G.A.M.K.); 6 FrieslandCampina, P.O. Box 1551, 3800 BN Amersfoort, The Netherlands; jeske.hageman@frieslandCampina.com; 7 Department of Human Nutrition, Faculty of Public Health, College of Medicine, University of Ibadan, Ibadan 200005, Nigeria; samuelfolake@gmail.com

**Keywords:** anaemia, malnourished, iron deficiency, iron deficiency anaemia, microbiota, Nigeria, toddler, multi-nutrient fortified dairy-based drink

## Abstract

Prevalence of anaemia among Nigerian toddlers is reported to be high, and may cause significant morbidity, affects brain development and function, and results in weakness and fatigue. Although, iron fortification can reduce anaemia, yet the effect on gut microbiota is unclear. This open-label randomised study in anaemic malnourished Nigerian toddlers aimed to decrease anaemia without affecting pathogenic gut bacteria using a multi-nutrient fortified dairy-based drink. The test product was provided daily in different amounts (200, 400 or 600 mL, supplying 2.24, 4.48 and 6.72 mg of elemental iron, respectively) for 6 months. Haemoglobin, ferritin, and C-reactive protein concentrations were measured to determine anaemia, iron deficiency (ID) and iron deficiency anaemia (IDA) prevalence. Faecal samples were collected to analyse gut microbiota composition. All three dosages reduced anaemia prevalence, to 47%, 27% and 18%, respectively. ID and IDA prevalence was low and did not significantly decrease over time. Regarding gut microbiota, *Enterobacteriaceae* decreased over time without differences between groups, whereas *Bifidobacteriaceae* and pathogenic *E. coli* were not affected. In conclusion, the multi-nutrient fortified dairy-based drink reduced anaemia in a dose-dependent way, without stimulating intestinal potential pathogenic bacteria, and thus appears to be safe and effective in treating anaemia in Nigerian toddlers.

## 1. Introduction

Anaemia, which is characterised by a haemoglobin level below 11.0 g/dL, continues to be a serious global public health problem that particularly affects young children and pregnant women [1]. The World Health Organization (WHO) reported that an estimated 42% of children aged <5 years are anaemic worldwide whereas the burden is even higher in Africa, reaching 62.3% [2]. Anaemia is associated with significant morbidity, affects normal brain development and function, and results in weakness and fatigue [2]. Literature [3,4,5,6] indicates that iron deficiency (ID), when serum ferritin levels are low, is the most common cause of anaemia [5,6,7] and a result of low iron intake and or iron malabsorption, decreased iron uptake into the blood, increased requirements, or blood loss.

Dietary iron absorption occurs almost exclusively in the duodenum, where it can be absorbed as haem, iron chelates, or as ferrous iron (e.g., from ferrous sulphate) [1,8]. In the body, the total amount of iron, and free iron, in particular, is guarded by a controlled absorption (via iron stores in the enterocyte), body iron status (via hepcidin blockade of iron transport into the blood), and by transport and storage proteins such as lactoferrin, transferrin, ferritin and hemosiderin in circulation and organs [9,10,11,12]. Synthesis of both hepcidin and ferritin (acute phase protein) is increased during infection and inflammation. In this way, the transport of iron into the circulation is limited and iron is sequestered in the reticuloendothelial system, which in the end may lead to anaemia [2,13,14]. Increased levels of ferritin, due to inflammation may overestimate iron status, and that is why the ferritin cut-off value for iron deficiency is increased when CRP is increased.

Multiple micronutrient powders (MNP) are strongly recommended in areas with anaemia prevalence in children of 20% or higher [15]. These supplements contain 10–12.5 mg of elemental iron, often supplied as ferrous fumarate (FeFum) or ferrous sulphate heptahydrate, per sachet [15]. A recent Cochrane review concluded that MNP is an effective intervention to reduce the risk of ID and anaemia in infants and young children, 6–23 months of age [16]. Lower iron doses have also been proved successful in increasing Hb. In a 9-month iron-fortified complementary foods intervention study, the efficacy of 2 mg of NaFeEDTA plus 3.8 mg of either FeFum or ferric pyrophosphate (FePP) was explored in toddlers (12-36 months of age) in a highly malaria-endemic region. Following the treatment (6 days/week), Hb increased in the FeFum group from 9.9 to 10.4 g/dL and in the FePP group from 9.6 to 10.5 g/dL [10]. Iron-deficiency anaemia (IDA), the most severe degree of ID [17], sharply decreased from 32.8–1.2% (FeFum) and from 23.6–3.4% (FePP) [18].

Besides MNP there is another way to introduce higher levels of iron in the diet of young children, via young child formula (YCF). In a study conducted in New Zealand and Australia, daily provision of 300 mL iron-fortified (1.7 mg/100 mL elemental iron [source not mentioned]) YCF significantly improved the iron status of children as compared to unfortified cow’s milk [19]. Iron-fortified YCF is also mentioned by the European Society for Pediatric Gastroenterology, Hepatology, and Nutrition (ESPGHAN) Committee on Nutrition as an efficacious option to increase iron status in children [20]. 

Although iron fortification has positive effects on anaemia and iron status, some studies indicate that oral iron may negatively affect the composition of the intestinal microorganisms (microbiota) [21,22]. Except for *Lactobacilli* and *Borrelia burgdorferi*, all microorganisms require iron to survive [8]. Many pathogens have acquired strong mechanisms for acquiring iron from the environment, e.g., by secreting iron chelators (e.g., siderophores) that facilitate the bacterial uptake of iron [22,23]. In contrast, beneficial commensal gut bacteria from the genera *Lactobacillus* and *Bifidobacterium* require little or no iron [22]. In the case of low iron intake, the availability of iron for the microbiota is even stimulated by bacterial metabolites (in particular produced by a few *Lactobacillus* strains) that reduce the absorption of iron by decreasing the activity of hypoxia-inducible factor (HIF-2α) in the enterocyte. HIF-2α regulates directly the three key intestinal iron transporters [24]. However, in case of sufficient iron intake, or the use of iron-fortified food and supplements, the amount of iron available for the microbiota is more than enough since absorption is relatively low (5–20%); typically, 20% of the iron is absorbed in the duodenum [6,7]. Meaning that most of the iron passes unabsorbed into the colon and may, in particular, stimulate (probably dose-dependently) the more pathogenic bacteria.

In 6-month-old Kenyan infants consumption of MNP with either 2.5 mg (NaFeEDTA) or 12.5 mg (FeFum) iron daily for 4 months adversely affected the gut microbiota composition [21]. For both fortifications, an increase in *Enterobacteriaceae* (including pathogenic *E. coli*) and a decrease in *Bifidobacteriaceae* abundances were observed. Furthermore, the incidence of diarrhoea that required treatment was increased in the higher iron dose group, as compared to no iron fortification (27.3% vs. 8.3%, respectively). This study indicates that iron fortification should be balanced: high enough to treat anaemia, but without affecting the gut microbiota. 

In the present study, a multi-nutrient fortified dairy-based drink was provided daily in different amounts (200, 400 and 600 mL, supplying 2.24, 4.48 and 6.72 mg of elemental iron, respectively, provided as ferrous sulphate) to anaemic, malnourished Nigerian toddlers (12–36 months of age) for 6 months. This study aimed to investigate the effect of different doses of multi-nutrient fortified dairy-based drink (including iron) on the reduction of anaemia prevalence in the target population without stimulating potential pathogenic bacteria in the gut.

## 2. Materials and Methods

### 2.1. Subjects and Study Design

In this three-arm, open (blind for biochemical analyses) randomised intervention trial, apparently healthy Nigerian toddlers (*n* = 184), but having mild-moderate anaemia (Hb ≥ 7.0 g/dL and ≤10.9 g/dL) and mild-moderate acute malnutrition (Height-for-age Z score (HAZ) and/or Weight-for-age Z score (WAZ) <−1 SD and >−3 SD), were recruited in Ijora-Badia community in Apapa-Iganmu Local Council Development Area (LCDA), Lagos, South-West Nigeria. Children with severe malnutrition and anaemia were excluded since they require additional measures such as hospital admission and blood transfusion. At birth, all subjects were born vaginally (since the way of birth may affect microbiota composition [25]) and term, and should be able to consume a maximum of 600 mL of product per day at the time of inclusion in this study. Children were not included when they (I) had a chronic or severe illness requiring hospitalisation and/or special treatment, (II) had a recent medical history (past 3 months) of serious infections, injuries and/or surgeries, (III) had any known allergies or intolerances to milk or milk ingredients, (IV) were predominantly breastfed toddlers, (V) consumed any other fortified foods or supplements, (VI) participated in micronutrient supplementation programs, (VII) participated in any other nutritional study in the last 6 months, (VIII) participated in another clinical study or received an investigational drug in the last 30 days, (IX) were likely to move within the period of intervention, (X) were related or employed by the sponsor or the university, (XI) used any prescription medications before and/or during the study period for more than or equal to two weeks. No restrictions were set for regular food intake.

For recruitment, all subjects gave their informed consent for inclusion before they participated in the study. All families that were permanent residents of Ijora-Badia and with toddlers were informed about the study by a mobilisation team working in the Ijora Badia community from the Lagos State Ministry of Health a few weeks before the commencement of the study. During information meetings, parents, or legal guardians of potential candidates (toddlers) were fully informed about the study, the requirements, and procedures, and all their questions were answered. At the screening, when a signed informed consent was obtained from parents or legal guardians, trained researchers verified age (by birth certificate confirmation or caregiver), and took anthropometric measurements (weight, height, waist-, head-, and mid-upper arm circumference). Eligible children were directly enrolled by trained researchers and randomly (computer-generated block-randomisation, based on the order of screening and stratified for gender and age (12–20–27, and 28–36 months of age)) assigned by the principal investigator to one of the three study groups, with an allocation ratio of 1:1:1. 

Following inclusion, study participants received deworming treatment (10 mg/kg bodyweight pyrantel pamoate), to assure that expected worm infections would not play a role during the study. Afterwards, baseline measurements were performed. Venous blood samples (10 mL) were taken for the assessment of iron status and inflammation parameters. Samples of faeces and early morning urine were collected before the start of the intervention. All measurements were repeated at the end of the 6 months intervention period. WHO Anthro 2007 [26] was used to generate z-score values for weight-for-age, height-for-age, weight-for-height, and BMI-for-age.

### 2.2. Ethics

The study was conducted in accordance with the Declaration of Helsinki, and the protocol was approved by the Ethics Committee of Lagos State University Teaching Hospital and the Lagos State Government (LREC/10/06/829). The trial was registered at ClinicalTrials.gov: NCT03411590.

### 2.3. Study Products

The three groups received a multi-nutrient fortified dairy-based drink (Peak 123, FrieslandCampina Wamco, Lagos, Nigeria), in amounts of 200, 400 or 600 mL per day. In the case of 400 and 600 mL, parents were requested to spread the portions (200 mL each) during the day. The time of intake was not monitored. The composition of the drink is shown in Table 1. The ingredient list is presented in Table S1. Airtight packed powder sachets (for 200 mL each) were delivered weekly to the families by trained researchers who also provided instructions for use. Consumption of test products started as soon as all baseline examinations were completed and baseline blood, early morning urine, and faecal samples were collected. In the case of twins and siblings, only the child who met the inclusion criteria participated in the study officially, however, the other child received the same treatment to prevent sharing and noncompliance with the study protocol. 

### 2.4. Blood Sampling and Sample Preparation

Venous blood sampling was performed in the morning between 9:00 and 11:00 a.m. at baseline and endline, obtaining a maximum of 10 mL blood, collected in 3 different types of test tubes: EDTA microtainer (4 mL), heparin gel microtainer (4 mL), and a serum microtainer (2 mL). The EDTA and heparin microtainers were kept at 4 °C and transferred (on ice) to a local laboratory on the day of collection. In the laboratory, tubes were directly centrifuged (HaematoSpin 1400, Hawksley, UK) at 3300 g for 15 min and the extracted EDTA and heparin plasma was pipetted into aliquots of 200 μL. Serum microtainers were kept at room temperature for at least 60 min to allow clotting. Clotted blood was centrifuged at 2000 g for at least 3 min and the extracted serum was pipetted into aliquots of 200 μL. All serum and plasma aliquots were stored at −20°C and transported on dry ice to the Amsterdam University Medical Center (Location Vumc, Amsterdam, The Netherlands) for biochemical analyses. 

### 2.5. Biochemical Parameters

Hb was determined in whole blood using the HemoCue Hb 301 system kit (HemoCue AB, Ängelholm, Sweden). Anaemia was defined as Hb < 11.0 g/dL [27]. CRP was determined in heparin plasma using an immunoturbidimetric assay on Roche/Hitachi Cobas c systems (measuring range: 0.3–350 mg/L; interassay CV < 20% in low CRP samples; repeatability CV < 5%, intermediate precision CV < 12%). Serum ferritin was determined in heparin plasma using an immunoturbidimetric assay on Roche/Hitachi Cobas c systems (measuring range: 5–1000 µg/L; interassay CV < 20% in low ferritin samples; repeatability CV < 10%, intermediate precision CV < 14%). CRP and ferritin concentrations were used to determine ID and IDA. In the present study, ID was defined as <12 µg/L serum ferritin when CRP ≤ 5 mg/L (no inflammation) or <30 µg/L serum ferritin when CRP > 5 mg/L (inflammation), and IDA as the combination of ID and anaemia [2,27]. Folate was analysed in heparin plasma using the Elecsys Folate III binding assay and Cobas-e immunoassay analyzer (Roche/Hitachi). Measuring range: 0.6–20.0 ng/mL or 1.36–45.4 nmol/L. Vitamin B12 was determined in serum using the Elecsys Vitamin B12 binding assay and Cobas-e immunoassay analyzer. Measuring range: 50.0–2000 pg/mL or 36.9–1476 pmol/L. The cut-off values for folate and vitamin B12 used in this study were 10 nmol/L and 150 pmol/L, respectively. 

### 2.6. Faecal Samples and Microbiota Analysis

Parents/caregivers were asked to collect 5-10 g early morning faeces in stool collection stubs with a spoon attached to the lid (Greiner Bio-One, Vilvoorde, Belgium), at baseline and after the intervention. The samples were brought to the collection centres by the parents and caregivers and stored at (5–7 °C) within 1 h, and then transferred on ice to the nearest freezer (−20 °C) that same day. Samples were transported to the laboratory (NIZO, Ede, the Netherlands) on dry ice, where faecal pathogenic *Escherichia coli* was determined using targeted qPCR (*n* = 88; 200 mL *n* = 26, 400 mL *n* = 27, 600mL *n* = 35). Furthermore, 16S rRNA gene sequencing was performed in a subset of 60 samples (200 mL *n* = 19, 400 mL *n* = 20, 600 mL *n* = 21) to determine faecal microbiota composition as previously described [28]. A full description of the materials and methods used for DNA extraction and gut microbiome analysis is available as Supplementary Text.

### 2.7. Urine Samples and Iodine Analysis

The iodine status of the children was assessed using spot urinary samples. The parents were asked to collect the child’s early morning urine (5 mL) into a 10mL universal laboratory bottle at baseline and after the intervention, before any food or drink consumption, for determining urinary iodine concentrations. The samples were brought to the collection centres by the parents and caregivers and stored at 5–7 °C within 1 h, and then transferred on ice to the nearest freezer (−20 °C). Samples were transported on dry ice to the Central Clinical Laboratory of the University Medical Center Groningen, the Netherlands. Iodine in urine was analysed using ICP-MS (Varian, Varian Inc., Palo Alto, USA; lowest level of quantification (LLOQ) 25 µg/L). The cut-off value used for iodine deficiency in this study was <100 ug/L [29]. 

### 2.8. Sample Size Calculation

Although the change in anaemia prevalence was the primary objective of the study, the sample size calculation was based on an expected change over time in Hb (baseline versus endline) as at baseline all included children were anaemic. The expected change in the 600 mL group (6.72 mg Fe/day) was 1.0 ± 1.5 g/dL, based on the finding of Sazawal et al., 2010 [30]. A total sample size of 35 toddlers per treatment arm was considered adequate to detect the estimated increase in Hb (two-sided-power: 80%; α = 0.05). To compensate for a possible drop-out of about 30%, the sample size was increased to 45 toddlers per treatment arm, however, during recruitment, 184 children were included in the study.

### 2.9. Statistical Analysis

All participants with at least weight measurement at baseline were included in the intention to treat (ITT) population. Subjects were included in the Per Protocol (PP) population when Hb measurements were available at both baseline and endline. Baseline subject characteristics of the three groups within the PP population were compared using the Pearson Chi-square test, Fischer’s exact test, independent t-test, one-way ANOVA, Mann–Whitney U test or Kruskal–Wallis H test depending on the type and distribution of the parameter. All analyses were performed using IBM SPSS Statistics version 24 (IBM Corp, Armonk, NY, USA). 

### 2.10. Evaluation of Biochemical and Related Parameters

The post-intervention prevalence of anaemia of the three groups was compared using a Pearson Chi-Square test, with post hoc pairwise comparisons using the z-test of two proportions with a Bonferroni correction. ANCOVA was used to determine the effect of the different interventions on Hb and ferritin, including baseline concentrations as a covariate. Post hoc analyses were performed with a Bonferroni adjustment. Differences in Hb and ferritin between baseline and endline within intervention groups were tested with either a paired t-test or Wilcoxon signed-rank test, depending on the distribution of the data. ID, IDA, and inflammation prevalence between groups were compared using Fisher’s Exact test. Within-group differences in ID, IDA, and inflammation prevalence were tested using McNemar’s test. A *p*-value < 0.05 was considered significant. All analyses were performed using IBM SPSS Statistics version 24 (IBM Corp, Armonk, NY, USA).

### 2.11. Evaluation of Faecal Microbiota

Between-group differences in alpha diversity (variance within a particular sample) and beta diversity (variation between samples; phylogenetic distance metric weighted UniFrac) were tested by the nonparametric Kruskal–Wallis test with Dunn’s posthoc test, as implemented in GraphPad Prism 5.01. Between-group differences of single taxa were assessed using the nonparametric Mann–Whitney U test with FDR correction for multiple testing unless stated otherwise. Comparisons of targets of our primary interest (*Lactobacillaceae, Bifidobacteriaceae, Enterobacteriaceae*) were not corrected for multiple testing. For comparisons of more than 2 groups, the nonparametric Kruskal–Wallis test with Dunn’s posthoc test was applied. In the longitudinal analysis, change of taxon relative abundance over time, 2log ratios were calculated, in which the relative abundance of a taxon at the second or later time point was divided by the relative abundance of the same taxon at an earlier time point. Ratios were compared among groups by Mann–Whitney U tests with FDR correction for multiple testing, and for comparisons of more than 2 groups by the nonparametric Kruskal–Wallis test with Dunn’s posthoc test. Redundancy analyses (RDAs) on the gut microbiota composition as assessed by 16S rRNA gene sequencing were performed in Canoco version 5.11 using default settings of the analysis type “Constrained”. Relative abundance values of genera or operational taxonomic unit (OTUs) were used as response data and metadata as an explanatory variable. For visualisation purposes, families (and not OTUs) were plotted as supplementary variables. Longitudinal effects of the intervention were assessed by calculating 2log ratios in which the relative abundance of an OTU or genus at endline was divided by the relative abundance of the same OTU or genus at baseline. These ratios were used as response variables in RDAs and were weighted based on the average relative abundance of each OTU or genus in all subjects. RDA calculates *p*-values by permutating (Monte Carlo) the sample status. qPCR gene copy counts of each target (total bacterial counts, EPEC *eaeA* gene, ETEC *lt* gene, ETEC *st* gene) were compared between test product dose groups by Kruskal–Wallis test with Dunn’s posthoc test and changes over time were calculated by subtracting the counts at endline from the counts at baseline. Pathogenic *E. coli* was defined as the sum of the gene copies of EPEC, ETEC lt and ETEC st.

## 3. Results

### 3.1. Baseline Characteristics

**Figure 1:**
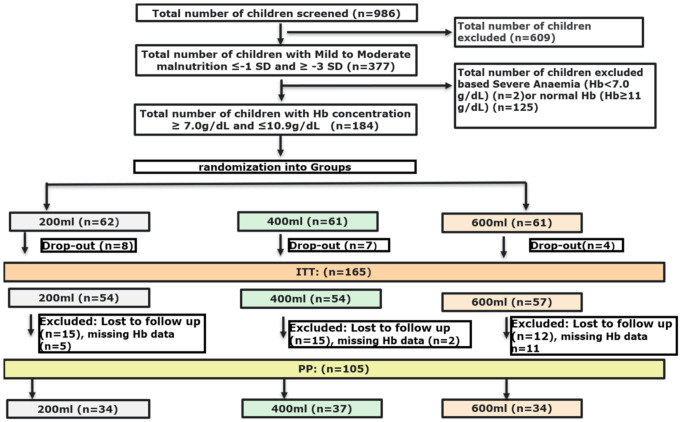
Flow-chart of screening and randomisation process.

In total 184 Nigerian toddlers were recruited, between February and June 2018, and allocated to one of the study groups: 200 mL *n* = 62, 400 mL *n* = 61, and 600 mL *n* = 61. Nine children without any anthropometric and/or no iron-status biochemical data at baseline, seven children who did not comply with the malnutrition criteria (false inclusions), and three children with very high ferritin but low CRP levels (suspected iron metabolic disease) were excluded from evaluation. Meaning that 165 children were finally included in the ITT analysis, as shown in Figure 1. We experienced a high loss to follow-up, mainly due to the traditional travelling of families during the festive periods such as Id el Kabir and Christmas (*n* = 42). For 18 toddlers Hb concentrations at endline were missing. This resulted in a population of 105 toddlers for PP analysis 105 subjects. The baseline characteristics of the three different intervention groups of the PP population are shown in Table 2. No significant differences were found between the three groups, except for height which was lower in the 600 mL group compared to the 200 mL group (*p* = 0.03). The baseline characteristics of both ITT and PP populations can be found in Table S2 and were comparable between populations except for inflammation prevalence, which was higher in the ITT population (30.6%) compared to the PP population (17.6%). 

### 3.2. Anaemia Prevalence and Hb

After the intervention, anaemia prevalence was reduced for all treatment groups, but the effect was significantly different between the treatments (*p* = 0.03), see Table 3. The intervention with 600 mL resulted in a significantly lower anaemia prevalence compared to the 200 mL intervention (*p* = 0.03). The anaemia prevalence of the 400 mL group was not different from the other groups. The intervention also improved Hb concentrations in each group (*p* < 0.0005), see Table 3. After adjustment for Hb concentration at baseline, there was a significant difference in post-intervention Hb concentrations between the three treatments (*p* = 0.005), being higher for the 400 and 600 mL groups as compared to the 200 mL group (*p* = 0.01 and *p* = 0.01, respectively). 

Median ferritin concentrations did not significantly increase during the intervention. Post-intervention concentrations were not different between treatment groups (*p* = 0.84). The CRP concentrations in the 600 mL group significantly decreased compared to baseline (median 0.7, IQR 1.7, *p* = 0.02). This effect was not found for the 200 and 400 mL groups (median 2.0, IQR 4.0 and median 2.1, IQR 3.1, respectively). A one-way ANCOVA, with baseline CRP as a covariate, did not show any difference in post-intervention CRP concentrations between the three groups (*p* = 0.19). Inflammation prevalence following treatment was not different between treatment groups (*p* = 0.27). Within treatments, there seemed to be a reduction in the 400 and 600mL groups (from 19.4% to 11.1% and from 15.2% to 6.1%, respectively), but this was not significantly different (*p* = 0.29 and *p* = 0.38, respectively). For the 200mL group, the inflammation prevalence at endline was 20.0%, not different from baseline (*p* = 1.00). 

### 3.3. Iron Deficiency and Iron-Deficiency Anaemia

The prevalence of ID at baseline was not statistically different between treatment groups (*p* = 0.25) (Table 4). As all children were anaemic at baseline, the results for IDA at baseline are similar to ID. Following treatment, a trend for a decrease in ID prevalence was found for the 200 mL group (*p* = 0.06). No significant differences were found in the 400 and 600 mL groups (*p* = 0.50 and *p* = 1.00 respectively). Taking the whole PP population into account (independent of consumption volumes), ID significantly reduced from 12.1% at baseline to 3.0% at endline (*p* = 0.04). Similar results were found for IDA. The iron status parameters of the iron and non-iron deficient toddlers at baseline are presented in Table S3. This indicates that the toddlers who were not iron deficient at baseline recovered faster from anaemia, as prevalence in the iron-deficient at baseline group prevalence after the intervention was 41.7%, while for the non-iron deficient at baseline the prevalence was 28.7% after the intervention.

### 3.4. Faecal Microbiota

For qPCR, 81 paired (base- and endline) samples, four single baseline and three single endline samples were studied. Faecal samples from other children were not suitable for analysis, due to insufficient amounts, or were missing. For 16S rRNA gene sequencing 34 paired samples, and 26 single endline samples were used. Based on quality control, one sample was removed because of a very low read count. On average 32,926 (25,930–48,354) bacterial 16S rDNA sequences per sample were analysed. The average gut microbiota composition at baseline is shown in Figure S1. There was no significant link between microbiota composition for any of the parameters gender, CRP, serum vitamin B12 and serum ferritin, as determined through RDA at both time points, i.e., they were not confounders. With regard to alpha diversity, no significant difference was found at baseline between the groups. At endline phylogenetic diversity was different between the groups (*p* = 0.0138) and lowest within the 400 mL group (*p* < 0.05) as compared to the 600 mL group (Figure S2). The change in diversity within individuals over time was not significantly different.

**Figure 2:**
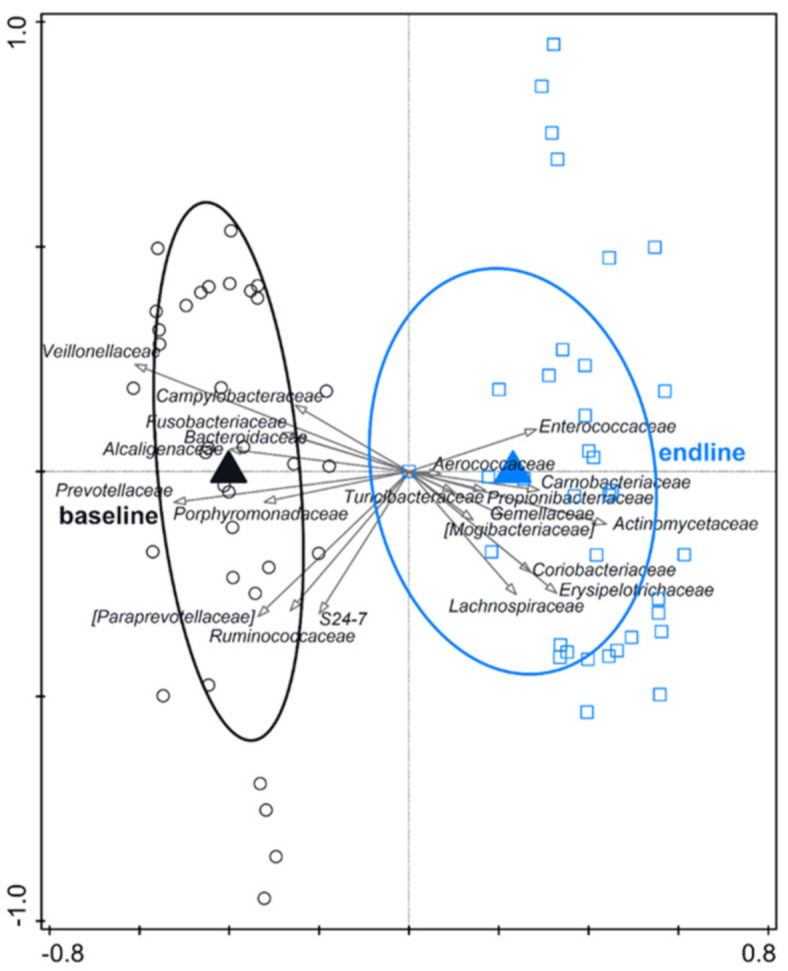
RDA on the operational taxonomic unit (OTU) level, assessing the effect of time (including test formula treatment) on gut microbiota composition. The covariance attributable to the subject was first fitted by regression and then partially out (removed) from the ordination. OTUs were used as response data and the time point was explanatory data, the bacterial families that contributed most were plotted supplementary. Variation explained by time point was 16.8%, *p* = 0.002.

RDA indicated a clear effect of time (variation explained by time point was 16.8%, *p* = 0.002) (Figure 2). Baseline samples were associated with e.g., *Veillonellaceae* and *Prevotellaceae*. Endline samples were associated with e.g., *Actinomycetaceae* and *Erysipelotrichaceae*, but not with *Enterobacteriaceae*.

**Figure 3:**
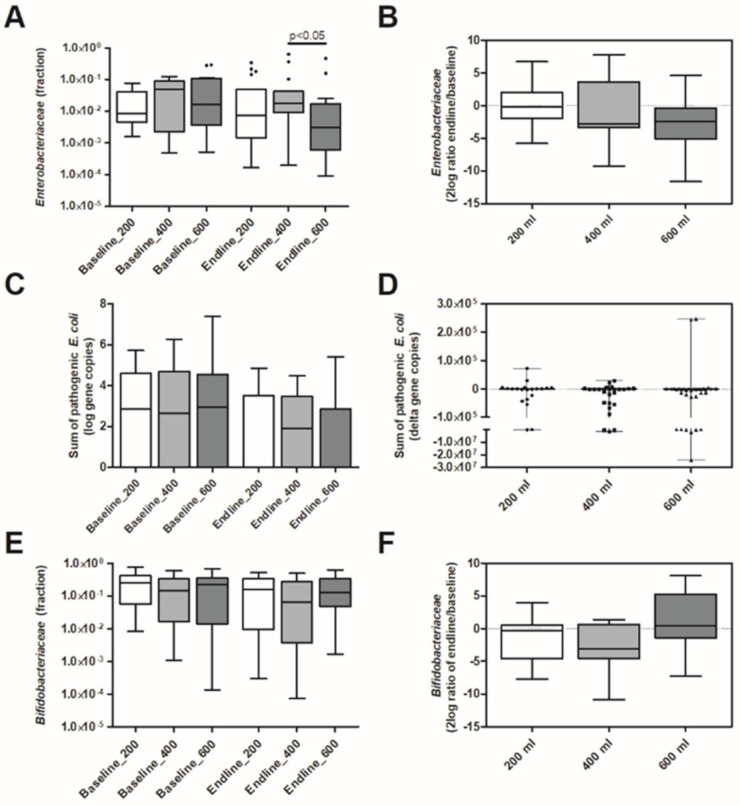
Effect of daily intakes of 200, 400, or 600 mL of YCF during 6 mo. on Enterobacteriaceae, pathogenic E. coli and Bifidobacteriaceae. Boxplots are displayed as Tukey whiskers. (**A**) Relative abundance of Enterobacteriaceae at baseline and endline. At endline relative abundance was significantly higher in the 400 mL group compared to the 600 mL group (*p* < 0.05 based on Dunn’s posthoc test). (**B**) Change in the relative abundance of Enterobacteriaceae over time (2log ratio of relative abundance at endline and baseline). (**C**) Abundance of pathogenic E. coli at baseline and endline (pathogenic E. coli represents the sum of the log gene copies of EPEC, ETEC lt and ETEC st). (**D**) Change in abundance of pathogenic E. coli over time (delta gene copies). (**E**) Relative abundance of Bifidobacteriaceae at baseline and endline. (**F**) Change in the relative abundance of Bifidobacteriaceae over time (2log ratio of relative abundance at endline and baseline).

Based on RDA at endline, there is a significant treatment effect (variation explained 2.2%; *p* = 0.006), but without a linear dose–response (Figure S3). The gut microbiota composition of toddlers on 400 mL appeared different compared with those on 200 or 600 mL. More specifically, *Enterobacteriaceae* were different between the groups (*p* = 0.041) at endline with a higher relative abundance associated with the 400 mL dose and lowest abundance with the 600 mL dose (*p* < 0.05) (Figure 3A). *Enterobacteriaceae* decreased over time (baseline to endline, Figure 3B), and the decrease was not significantly different between the groups. The higher level of *Enterobacteriaceae* at endline in the 400 mL group was reflected in a higher ratio of *Enterobacteriaceae-Bifidobacteriaceae* as compared to the 600 mL group (*p* < 0.01) (Figure S4). The relative abundance of *Bifidobacteriaceae* at endline and change over time did not differ between groups (Figure 3E,F). Pathogenic *E. coli* qPCR, targeted at EPEC ETEC *lt* and ETEC *st*, revealed no differences between the groups (Figure S5). At endline the sum of pathogenic *E. coli* (sum of EPEC, ETEC *lt* and ETEC *st*) was slightly lower compared to baseline, but the change was not significantly different between the test product doses (Figure 3C,D). 

## 4. Discussion

This study showed that daily consumption of different doses (200, 400 or 600 mL) of multi-nutrient fortified dairy-based drink for 6 months dose-dependently reduced anaemia prevalence in anaemic malnourished Nigerian children aged 1–3 years. Hb concentrations increased with all interventions, but the effect was more pronounced in the 400 and 600 mL groups as compared to 200 mL. Furthermore, this study showed that daily consumption of 200–600 mL multi-nutrient fortified dairy-based drink did not cause an increase in the potentially pathogenic gut bacteria. 

### 4.1. Anaemia Prevalence Reduction

In the current study, anaemic toddlers received different dosages of iron fortification (2.24, 4.48, and 6.72 mg/day) in the form of a multi-nutrient fortified dairy-based drink. All three dosages reduced anaemia prevalence from 100% at baseline to 47%, 27% and 17.6%, respectively, at endline. A similar trial conducted by Rivera et al. studied the effect of 400 mL iron-fortified milk (5.28 mg elemental iron from ferrous gluconate), administered as two 200 mL servings per day for 12 months, on anaemia and ID prevalence in Mexican children aged 12–30 months with mild-to-moderate anaemia (Hb < 11g/dL) [31]. After 6 months of intervention, anaemia prevalence in the fortified milk group decreased from 44.5% at baseline to 12.7% (reduction of 72%), while the nonfortified milk group (0.16 mg Fe/400 mL;) showed a 54% reduction in anaemia prevalence, from 42.6% to 19.7% [31]. The effect of the iron-fortified milk is in line with the results of the 400 mL group in the present study, showing a 73% reduction in anaemia prevalence, whereas the 600 mL (6.7 mg iron) group showed an 82.5% reduction. Surprisingly, the effect of unfortified milk in the study of Rivera et al. is equal to the effect as seen in our 200 mL group. Although intervention durations were different, 12 versus 6 months in the present study, in general, it is assumed that the contribution of cow’s milk to total iron intake is not relevant. Moreover, the consumption of cow’s milk by toddlers is reported to be associated with declining iron stores [32]. Rivera et al. did not discuss this finding in the nonfortified group in detail but the improvement suggests an effect of other nutrients, possibly protein coming with the milk [33], change in diet, or other features associated with the program. De-worming, which is known to reduce anaemia, did not take place in the Mexican study [31]. 

### 4.2. ID and IDA Not the Most Important Causes of Anaemia

ID is present when serum ferritin levels are lower than the defined cut-off values: <12 µg/L when CRP ≤ 5 mg/L or <30 µg/L when CRP > 5 mg/L for children 0–59 months [2]. Low Hb (<11 g/dL) in combination with ID results in IDA. In the present study, with only anaemic children enrolled, the mean prevalence of ID and IDA in the PP population at baseline were similar, on average 12.1%. These prevalences reduced to 3% after 6 months of intervention. This low ID outcome conflicts with the general statement that ID is the major cause of anaemia [34]. However, studies in Akwa Ibom State (Nigeria) and Côte d’Ivoire also indicated a low ID prevalence in anaemic children of 6.7% and 13.2%, respectively [35,36]. In a Mexican study, ID prevalence at baseline was 37% and 30% in the nonfortified and fortified group, respectively [31]. Although ID in the Mexican study contributed more to the cause of anaemia as compared to our study, it was not the major contributor to anaemia either. Other reported major causes of anaemia are concurrent infection (including parasites), RBC- and/or iron metabolic diseases, acquired- and autoimmune-haemolytic anaemia, hypersplenism, transient erythroblastopenia, hypothyroidism, vitamin B12 or folate shortcomings, and protein deficiency [33,37]. From this list, it is unlikely that vitamin B12 and folate deficiencies were major causes in the present study since their prevalences at baseline were 3% and 12%, respectively. Additionally, hypothyroidism can be excluded since urinary iodine excretion was 2–3 times higher than the cut-off value of 100 μg/L: being >300 μg/L in most children at baseline (median 309 μg/L, range: 22–5622). Additionally, protein deficiency is a possible cause of non-ID anaemia as protein is necessary for Hb synthesis. This type of anaemia is reversible by feeding a complete protein [33] such as the cow’s milk protein in the multi-nutrient fortified dairy-based drink in the present study. Therefore, the observed effect of this study might be partly due to increased protein intake. Worm infestations are associated with (ID) anaemia and these infections probably are tolerated by the immune system meaning that parameters of inflammation will not increase (in contrast, worm infections may be anti-inflammatory) [38,39,40]. The effect of anthelmintic treatment in toddlers is unknown but in a literature review and meta-analysis conducted on the effects of deworming on child and maternal health, deworming did not show consistent benefits for indicators of mortality, anaemia, or growth in children younger than five or women of reproductive age [41]. Before starting the intervention of the present study all children received deworming treatment. As no control group was included, it cannot be excluded that the observed reduction in anaemia prevalence was partly related to this treatment. However, as anaemia prevalence was further reduced in the 400- and 600-mL groups compared to the 200 mL group, the multi-nutrient fortified dairy-based drink seems to affect anaemia on its own. In addition, malaria is a possible cause of anaemia in children, without causing ID, and supplementation with iron following malaria has been reported to promote recovery from anaemia in 4-6 weeks [42]. Malaria is endemic in Nigeria and Lagos State. [43]. A review article on the epidemiology of malaria in endemic areas shows an illustration of the start/end of malaria transmission season in Lagos being from April till December [44] which perfectly coincides with the intervention period of the present project. This could imply that malaria at least in part was a cause of anaemia without ID. When antimalaria drugs were prescribed (not recorded in this study but assumed by a local paediatrician), recovery might have been stimulated by iron supplementation. Taken together, several factors or combinations of factors other than ID may cause anaemia. For the present study, anthelmintic infection and malaria are plausible factors, and their treatments may have contributed to anaemia reduction. However, as a dose–response effect was observed in this study it can be concluded that the multi-nutrient fortified dairy-based drink relevantly contributed to the reduced anaemia. The total composition of multi-nutrient fortified dairy-based drink, but in particular the combination of a good available iron source (ferrous sulphate), sufficient vitamin C (to further stimulate iron availability), and likely also the cow’s milk protein, probably contributed to this beneficial effect on anaemia [33,45,46]. 

### 4.3. Effect of Multi-Nutrient Fortified Dairy-Based Drink on Gut Microbiota

Food fortification programs are usually considered the most cost-effective and sustainable approach to combat iron deficiency, especially in sub-Saharan African countries. However, most of the iron will not be absorbed and passes into the colon [46,47] where it may be used in particular by potential pathogenic gut bacteria such as *Salmonella*, *Shigella* or pathogenic *Escherichia coli* [48,49]. This adverse effect has been shown in two double-blind randomised controlled trials. In 6-month-old Kenyan infants consuming iron-fortified (2.5 or 12.5 mg) maize porridge daily for 4 months, the growth of potentially pathogenic bacteria was stimulated whereas the relative abundance of beneficial ‘barrier’ *bifidobacteria* decreased [21]. This shift towards a potentially more pathogenic profile was associated with intestinal inflammation, as was reflected in an increased faecal calprotectin concentration [21]. Furthermore, the increase in *enterobacteria* correlated with an increase in faecal calprotectin concentration [49]. Additionally, in a 6-months, randomised, double-blind, controlled trial, in 6–14-y-old Ivorian children, consumption of iron-fortified biscuits (20 mg/day, 4 times/week) increased the number of faecal *enterobacteria* and decreased the number of *lactobacilli*. At least one study has shown that the negative effect of iron on gut microbiota can be corrected by the concurrent addition of galactooligosaccharides [28]. Two additional fortification trials in infants with an MNP containing 12.5 mg FeFum have raised safety concerns: in Ghana, there was an increased rate of hospitalisation and based on data from the outpatient register, 83% of the additional cases in the iron group were due to diarrhoea, but this was not significant [50], and in Pakistan, a small but significant increase in overall diarrhoea prevalence, bloody diarrhoea, and respiratory illness was reported [51]. In the present study, we did not find any adverse effects of the intervention on or related to the gut microbiota, such as diarrhoea. *Enterobacteriaceae* relative abundance decreased over time and the magnitude of decrease was not different between groups. Moreover, after the intervention, the lowest abundance of *Enterobacteriaceae* was found in the 600 mL group. Furthermore, pathogenic *E. coli* decreased over time and did not differ between groups, and the slight decrease in *Bifidobacteriaceae* over time was not different between the groups. Based on this information, it appears that feeding a multi-nutrient fortified dairy-based drink, containing low levels of relatively high bioavailable iron per serving (iron sulphate, vitamin C, no phytic acid) [46,47] is unlikely to significantly disturb the gut microbiota in toddlers. The food matrix may have contributed to the nonobserved adverse effect, as well as the relatively small amount of iron provided per serving. Whether and how high thresholds are at which iron disrupts gut microbiota should be investigated. 

### 4.4. Strengths and Limitations

By using anaemia as an inclusion criterion, the effect on improving anaemia could be studied. The omission of a placebo group is a limitation, such that the effect on anaemia cannot be attributed to the multi-nutrient dairy-based drink alone. A placebo group would have been very helpful in determining the effect of deworming on anaemia prevalence. Furthermore, as the morbidity profile of the study subjects was not monitored, we do not know whether malaria might have played a role, and how the iron doses affected malaria recovery or severity.

The loss of quite a lot of children for follow-up (*n* = 42) was a pity and possibly could have been prevented in part when midpoint follow-ups would have been organised. We cannot exclude loss of information due to faecal 16S rRNA analysis in only a subgroup of children. For the purpose of the study, however, the analysis appears to be sufficient. 

Since medically diagnosed allergies, not having anaemia, intolerances to milk or milk ingredients were exclusion criteria, and children were recruited from a poor environment, study results cannot directly be generalised to children with different characteristics. 

A strength of this study is the food-based dose–response approach, showing that already 200 mL of product improves anaemia. Therefore, this amount could be a good starting point when discussing the possibilities of cheaper though effective products for those at the bottom of the pyramid. 

## 5. Conclusions

This study shows that daily consumption of 200–600 mL of iron-fortified multi-nutrient fortified dairy-based drink reduces anaemia without stimulating potential pathogenic gut bacteria in Nigerian toddlers. Although the main cause of anaemia in the study population is not clear (protein deficiency, malaria or worm infestations might be contributors, besides a minor role for ID), the study shows a dose–response effect of the multi-nutrient fortified dairy-based drink in reducing anaemia which is likely attributable to the well-available iron and possibly also to the high-quality protein. The finding that 200 mL of the multi-nutrient dairy-based drink (2.24 mg iron daily) already results in a relevant improvement of anaemia in 6 months is of interest since this amount is more affordable, for many households, than 400 or 600 mL. 

## Figures and Tables

**Table 1:** Composition of the multi-nutrient fortified dairy-based drink intervention product in the volumes of 200, 400 and 600 mL.

Nutrient	Unit	Per 200 mL	Per 400 mL	Per 600 mL
Energy	kcal	149	297	446
Protein	g	5	11	16
Carbohydrates	g	20	41	61
Sucrose	g	2.9	5.8	8.7
Lactose	g	7	14.5	21.7
Fat	g	5	10	15
DHA	mg	14	28	42
Calcium	mg	188	376	564
Phosphorus	mg	152	304	455
Potassium	mg	244	488	733
Magnesium	mg	17	33	50
Sodium	mg	63	125	188
Iron	mg	2.24	4.48	6.72
Copper	ug	58	116	173
Zinc	mg	1	2	3
Iodine	ug	40	79	119
Selenium	ug	3.6	7.3	11
Vitamin A	ug-RE	128	255	383
Vitamin D3	ug	2	4	6
Vitamin E	mg	3	5	8
Vitamin B1	ug	155	310	465
Vitamin B2	ug	158	317	475
Vitamin B6	ug	157	314	470
Folic acid	ug	24	48	71
Vitamin B12	ug	0.4	0.8	1,2
Vitamin K1	ug	9.2	18.5	28
Biotin	ug	5.3	10.6	16
Niacin	mg	2.0	4.0	6
Pantothenic acid	mg	0.7	1.3	2
Vitamin C	mg	38	76	114

**Table 2:** Baseline characteristics of the three intervention groups (PP population).

	200 mL	400 mL	600 mL	*p*-Value
*n*	34	37	34	
Age (months)	20.0, 8.5	20.0, 7.5	18.0, 8.5	0.48 °
Gender (boys/girls) (%)	47.1/52.9	51.4/48.6	38.2/61.8	0.53 ^#^
Social class (upper/middle/lower) (%)	0.0/21.2/78.8	2.8/19.4/77.8	0.0/17.6/82.3	0.57 ^#^
Religion (Muslim/Christian) (%)	72.7/27.3	63.9/36.1	70.6/29.4	0.71 ^#^
Weight (kg)	9.2, 2.3	8,9, 1.1	8.7, 1.8	0.42 °
Height (cm)	78.9 ± 5.5 ^a^	78.2 ± 4.8 ^a,b^	75.8 ± 4.4 ^b^	0.03 *
Weight for age Z-score	−1.78 ± 0.60	−1.71 ± 0.55	−1.78 ± 0.54	0.84 *
Height for age Z-score	−1.60 ± 0.61	−1.74 ± 0.53	−1.96 ± 0.62	0.06 *
Weight for height Z-score	−1.34 ± 0.77	−1.15 ± 0.72	−1.07 ± 0.72	0.30 *
Hb (g/dL)	10.4, 0.8	10.1, 1.7	10.0, 1.6	0.08 °
Ferritin (µg/L)	37.6, 36.9	35.7, 46.9	38.2, 52.1	0.83 °
CRP (mg/L)	1.7, 5.6	2.1, 7.2	2.1, 4.8	0.60 °
Inflammation prevalence (%)	18.2	19.4	15.2	0.89 ^#^
Vitamin B12 deficiency (%)	0.0	7.1	0.0	0.32 *
Folate deficiency (%)	9.1	8.0	21.1	0.40 *
Iodine (µg/L)	268.3, 405.7	327.6, 458.5	315.8, 467.5	0.99 °

Data are presented as median, IQR, percentages, or mean ± SD. Output of ° Kruskal–Wallis H test, ^#^ Chi-square test, or * one-way ANOVA. Different letters in superscript (a and b) indicate differences between treatment groups.

**Table 3:** Anaemia prevalence (%), Hb (mean ± SD) and ferritin concentrations (median, IQR) of the different treatment groups after 6 months of daily consumption of the multi-nutrient fortified dairy-based drink.

	200 mL	400 mL	600 mL	Treatment Effect *p*-Value
Anaemia prevalence (%)	47.1 ^a^	27.0 ^a,b^	17.6 ^b^	0.03
Hb (g/dL)	11.2 ± 0.9 ^a,^*	11.6 ± 0.9 ^b,^*	11.7 ± 1.0 ^b,^*	0.005
Ferritin (µg/L)	37.0, 19.3	34.7, 35.1	39.5, 28.4	0.84

* Significantly higher compared to baseline concentration (*p* < 0.05). Chi-square test was used for anaemia prevalence, one-way ANCOVA for Hb and ferritin with corresponding baseline concentrations taken along as covariate. In contrast to baseline concentrations, Hb at endline was normally distributed, and although this was not the case for ferritin ANCOVA was considered robust enough to apply for ferritin as well. Within treatment groups, concentrations before and after the intervention were compared using either paired t-test or Wilcoxon signed-rank test. Different letters in superscript (a and b) indicate differences between treatment groups.

**Table 4:** Prevalence of iron deficiency (ID) based on different ferritin cut-off levels depending on CRP levels [27], before and after the 6-month daily consumption of different amounts of multi-nutrient fortified dairy-based drink.

		200 mL	400 mL	600 mL	*p*-Value *
**ID prevalence**	**pre**	20.0%	11.1%	6.1%	0.25
	**post**	0%	2.8%	6.1%	0.53

* The prevalence between study groups has been compared with Fisher’s exact test. Iron deficiency (ID): <12 µg/L serum ferritin when CRP ≤ 5 mg/L or <30 µg/L serum ferritin when CRP > 5 mg/L.

## Data Availability

The data in this study are not publicly available but could be requested from the corresponding author.

## Supplementary Materials

The following are available online at https://www.mdpi.com/article/10.3390/nu13051566/s1*:* Supplemental text: Faecal samples and microbiota analysis; Table S1: List of ingredients; Table S2:Baseline characteristics of the ITT and PP population, Table S3: Anaemia prevalence (%), Hb and ferritin concentrations (median, IQR) of iron deficient and non-iron deficient toddlers, Figure S1: Average gut microbiota composition of the studied toddler population at baseline, from phylum to genus level; Figure S2: Alpha diversity (PD whole tree metric) in the multi-nutrient fortified dairy-based drink dose groups at baseline and endline; Figure S3: Redundancy analysis (RDA) on the OTU level, assessing the effect of test product dose on gut microbiota composition at endline; Figure S4: Enterobacteriaceae/Bifidobacteriaceae ratio in the test product dose groups at baseline and endline; Figure S5: Effect of daily intakes of 200, 400, or 600 mL of test product during 6 months on total bacterial counts (A) and pathogenic E. coli; EPEC (B), ETEC_lt (C), ETEC st (D) at baseline and endline. The test product was a multi-nutrient fortified dairy-based drink.
