# Supplementary material for: Multi-Nutrient Fortified Dairy-Based Drink Reduces Anaemia without Observed Adverse Effects on Gut Microbiota in Anaemic Malnourished Nigerian Toddlers: A Randomised Dose–Response Study
Source: Nutrients. 2021 May 6;13(5):1566. doi: 10.3390/nu13051566 (PMC8148581; doi:10.3390/nu13051566)
Supplement: Supplementary file 1 [file nutrients-13-01566-s001.zip › nutrients-1143747-supplementary.pdf]

**Supplemental text: Faecal samples and microbiota analysis**

Faecal samples were collected (5-10 g) in stool collection stubs with a spoon attached to the lid (Greiner Bio-One, Vilvoorde, Belgium), at the start and the end of the intervention. The samples were stored at a cold temperature within 1 hour, and transported on ice to the nearest freezer (-20°C) that same day. Samples were shipped on dry ice with temperature control to The Netherlands, where faecal microbiota was studied in a sub-sample of children (n= 88; group A n=26, group B n=27, group C n=35), by NIZO (Ede, The Netherlands) using 16S rRNA gene sequencing (in 60 samples; group A n=19, group B n=20, group C n=21) and targeted qPCR (in 88 samples).

**Bacterial DNA extraction, PCR amplification and 16S rRNA gene Illumina sequencing**

Faecal samples were first thawed at 4 °C. Then in a 2.0 mL screw-cap tube containing 0.5 g of 0.1 mm sterilized zirconia beads, 250 (± 10%) mg of faeces and 700 µL S.T.A.R. buffer (Roche, Indianapolis, IN, USA) were added. The FastPrep instrument (MP Biomedicals, Santa Ana, CA, USA) was used for lysis at 5.5 m/s for 3 times 1 min at room temperature. Thereafter samples were incubated while shaking at 100 rpm and 95 °C for 15 min. The samples were then centrifuged at 16000 g for 5 min at 4 °C. The collected supernatant was kept on ice, while another lysis round as described above, except that only 350 µl S.T.A.R. buffer was added, was done with the remaining stool pellet. The supernatant kept on ice was then pooled with the supernatant from the second lysis round. Purification of DNA was performed on the automated Maxwell instrument (Promega, Madison, WI, USA) by applying the Maxwell 16 Tissue LEV Total RNA Purification Kit (Promega) according to the manufacturer's instructions. To the first well of the Maxwell cartridge 250 µL of the supernatant was added and finally, DNA was eluted with 50 µL of RNAse/DNAse free water.

Using a 2-step PCR, barcoded amplicons from the V3–V4 region of 16S rRNA genes were generated (see library PCR below for a description of second PCR step). For initial amplification of the V3–V4 part of the 16S rRNA we used universal primers with the following sequences: forward primer, '5-*TCGTCGGCAGCGTCAGATGTGTATAAGAGACAG***<u>CCTACGGGAGGCAGCAG'</u>** (broadly conserved bacterial primer 357F in bold and underlined); reverse primer, '5-*GTCTCGTGGGCTCGGAGATGTGTATAAGAGACAG***<u>TACNVGGGTATCTAAKCC</u>**' (broadly conserved bacterial primer 802R (with adaptations) in bold and underlined), appended with Illumina adaptor sequences (in italics). The PCR amplification mixture contained: 1 µL faecal sample DNA, 1 µL barcoded forward primer (10 µM), 14 µL master mix (1 µL KOD Hot Start DNA Polymerase (1 U/µL; Novagen, Madison, WI, USA), 5 µL KOD-buffer (10×), 3 µL $MgSO_4$ (25 mM), 5 µL dNTP mix (2 mM each)), 1 µL (10 µM) of reverse primer and 33 µL sterile water (total volume 50 µL). PCR conditions were: 95 °C for 2 min followed by 30 cycles of 95 °C for 20 sec, 55 °C for 10 sec, and 70 °C for 15 sec. We then purified the approximately 500 bp PCR amplicons using the MSB Spin PCRapace kit (Invitek, Berlin, Germany).

For the library PCR step in combination with sample-specific barcoded primers, purified PCR products were shipped to BaseClear BV (Leiden, The Netherlands). PCR products were checked on a Bioanalyzer (Agilent) and quantified. This was followed by multiplexing, clustering and sequencing on an Illumina MiSeq with the paired-end (2x) 300 bp protocol and indexing. The sequencing run was analysed with the Illumina CASAVA pipeline (v1.8.3) with de-multiplexing based on sample-specific barcodes. From the raw sequencing data, we removed the sequence reads of too low quality (only "passing filter" reads

were selected) and discarded reads containing adaptor sequences or PhiX control with an in-house filtering protocol. On the remaining reads, a quality assessment was performed using FASTQC version 0.10.0. (http://www.bioinformatics.babraham.ac.uk/projects/fastqc/)

***Table 1:*** **Detection and quantification of pathogenic *E. coli* by qPCR**

The following quantitative real-time polymerase chain reaction (qPCR) assays were applied on isolated DNA from faecal samples:

| Bacterial target | Gene | Primers | reference |
|---|---|---|---|
| EPEC | *eaeA* | Fwd: 5′- GCCTTCATCATTTCGCTTTC′<br>Rev: 5′- GCCTTCATCATTTCGCTTTC′ | [1] |
| ETEC | In-house method; *st* and *lt* genes | Fwd *stla:* 5′ TTTCCCCTCTTTTAGTCAGTCAA′<br>*Fwd stlb:* 5′ TGCTAAACCAGTAGAGTCTTCAAAA′<br>Rev *stl*: 5′- GCAGGATTACAACACAATTCACAGCAG′<br><br>Fwd *lt*: 5′- TCTCTATGTGCATACGGAGC′<br>Rev *lt*: 5′- CCATACTGATTGCCGCAAT′ | [1] |
| Total counts | In-house method; 16S rRNA gene | Fwd: 5′- CGGTGAATACGTTCYCGG′<br>Rev: 5′- GGWTACCTTGTTACGACTT′ | [2] |

**Validation of the qPCR assays**

The *E. coli* qPCR assays were adapted from [1] and were first validated in a faecal matrix background (i.e. total microbial DNA isolated from faecal samples) using the following strains of *E. coli*:

- DSM8699 (EPEC)
- ATCC43887 (EPEC)
- DSM10937 (ETEC)
- H10407 (ETEC)

The following control samples were implemented for the validation:

- Target gene amplified DNA product (using bacterial genomic DNA);
- Faecal DNA (isolated from an adult faecal sample, diluted 10-fold) spiked with target gene amplified DNA product (positive control) (to determine potential inhibition of the assay);
- Purified water (negative control);
- Non-spiked faecal DNA (diluted 10-fold, negative control);
- Faecal DNA spiked with chromosomal DNA from *Campylobacter coli* DSM4689, *Campylobacter jejuni* DSM4688 (ATCC 35560), *Clostridium difficile* C630derm, *Clostridium perfringens* SM101,

*Klebsiella pneumoniae* DSM30104 (ATCC 13883), *Salmonella enterica* DSM17058 (LT2) (negative control) (specificity testing).

The slope, correlation coefficient and PCR efficiency were determined using values of three calibration curves on one plate. The $Cq_{min}$ and $Cq_{max}$ values (minimal and maximal number of amplification cycles) for each assay were determined using these calibration curves. Accuracy and intermediate precision of the assays were determined using three replicate measurements of three different dilutions (10x, 100x and 1000x) on three replicate plates. Accuracy was calculated as the difference between the experimentally measured value and the true value and is indicated in fold-change differences. The intermediate precision was calculated as a measure of the variation between plates using average and standard deviation values of quadruple measurements in two dilutions (100x and 1000x) of faecal matrix DNA background with spiked target gene amplified product in six concentrations ($10^2$ – $10^7$ copies/mL). The limit of detection and quantification (LOD/LOQ) was estimated using the lowest and the highest reliable Cq values of the standard curves of 10 replicate measurements. Any reliable measurement obtained with a value above 1 but below the LOD was still interpreted to reflect a probable quantification of the bacterial DNA present in the total DNA isolated from the faecal sample. Because the value is a true value (>1) but below the LOD, the value was included in the analyses, but renumbered to ½ LOD

***Table 2:* Validation values for three qPCR assays in a faecal matrix background.**

The following criteria were adjusted for acceptance of the validation outcome: slope; -3.1 to -3.8 (perfect slope is -3.3), correlation coefficient ($R^2$); ≥0.98 (perfect $R^2$ is 1.0), PCR efficiency; 90 – 110% (perfect is 100%), accuracy; <10-fold (perfect is 1.0-fold), precision; <35%.

| | EPEC | ETEC lt | ETEC st |
|---|---|---|---|
| Linear dynamic range | 1E8 – 1E1 | 1E8 – 1E1 | 1E8 – 1E1 |
| Slope | -3.371 | -3,450 | 3,312 |
| Correlation coefficient ($R^2$) | 0.982 | 0,993 | 0.987 |
| $Cq_{min}$ and $Cq_{max}$ | 9.4 - 33 | 11.1 - 35 | 10 – 32,9 |
| PCR efficiency | 98% | 95% | 100% |
| Accuracy | 1.2 | 2.3 | 1.3 |
| Intermediate precision | 11 – 34% | 10 – 25% | 5 – 33% |
| LOD/LOQ | $10^2$ copies/μL (=750 copies/mg) | $10^2$ copies/μL (=750 copies/mg) | $10^2$ copies/μL (=750 copies/mg) |

No positive signals >LOD were obtained against negative control species for any of the assays, meaning that the qPCR assays are specific against *E. coli* EPEC and ETEC.

***Table 3:* Execution of the qPCR assays**

The following PCR conditions were applied on three dilutions (10x, 100x and 1000x) of total microbial DNA isolated from faecal samples, in single measurements:

| Bacterial target | $T_{ann}$ | Primer dilution | Mastermix | Amplification curve slope ($\Delta C_q$) limits |
|---|---|---|---|---|
| EPEC<br>*eaeA* gene | 60 ˚C | Fwd: 200 nM<br>Rev: 200 nM | Primer + SYBR Green | Min: 2.2<br>Max: 4.6 |
| ETEC<br>*st* gene<br><br>*lt* gene | 60 ˚C<br><br>60 ˚C | Fwd: 250 nM<br>Rev: 250 nM<br>Fwd: 150 nM<br>Rev: 150 nM | Primer + SYBR Green<br>Primer + SYBR Green | Min: 2.2<br>Max: 4.6<br>Min: 2.2<br>Max: 4.6 |
| Total counts<br>16S rRNA gene | 56°C | Fwd: 500 nM<br>Rev: 500 nM | Primer + SYBR Green | Min: 2.2<br>Max: 4.6 |

Values were deemed reliable when within the $Cq_{min}$ and $Cq_{max}$ values of the assay (Table XXX) and within the ΔCq limits (2.2 – 4.6). The total number of copies per µl was calculated using the standard curve of the assay and used for calculation of the total number of copies of target-specific DNA present in the total microbial DNA isolated from the faecal samples using the following formula: (copies per µl/2)*200/250 = copies per mg.

**16S rRNA gene sequence analysis, qPCR analysis and statistics**

16S rRNA gene sequences were analyzed using a workflow based on Qiime 1.8 [3]. We performed operational taxonomic unit (OTU) clustering (open reference), taxonomic assignment and reference alignment with the pick_open_reference_otus.py workflow script of Qiime, using uclust as clustering method (97% identity) and GreenGenes v13.8 as a reference database for taxonomic assignment. Reference-based chimera removal was done with Uchime[4]. The RDP classifier version 2.2 was performed for taxonomic classification [5](ref Cole). Statistical tests were performed as implemented in SciPy (https://www.scipy.org/), downstream of the Qiime-based workflow.

We tested for between-group differences in alpha diversity and beta diversity (phylogenetic distance metric weighted UniFrac) by the non-parametric Kruskal-Wallis test with Dunn's posthoc test, as implemented in Graphpad Prism 5.01. Between-group differences of single taxa were assessed using the non-parametric Mann-Whitney U test with FDR correction for multiple testing; unless stated otherwise. Comparisons of targets of our primary interest (*Lactobacillaceae*, *Bifidobacteriaceae*, *Enterobacteria*ceae) were not corrected for multiple testing. For comparisons of more than 2 groups, the non-parametric Kruskal-Wallis test with Dunn's posthoc test was applied. In the longitudinal analysis, change of taxon relative abundance over time, 2log ratios were calculated, in which the relative abundance of a taxon at the second or later time point was divided by the relative abundance of the same taxon at an earlier time point. Ratios were compared among groups by Mann-Whitney U tests with FDR correction for multiple testing, and for comparisons of more than 2 groups by the non-parametric Kruskal-Wallis test with Dunn's posthoc test.

We performed redundancy analyses (RDAs) on the gut microbiota composition as assessed by 16S rRNA gene sequencing in Canoco version 5.11 using default settings of the analysis type

“Constrained[6]”. Relative abundance values of genera or OTUs were used as response data and metadata as an explanatory variable. For visualization purposes, families (and not OTUs) were plotted as supplementary variables. Longitudinal effects of the intervention were assessed by calculating 2log ratios in which the relative abundance of an OTU or genus at endline was divided by the relative abundance of the same OTU or genus at baseline. These ratios were used as response variables in RDAs and were weighted based on the average relative abundance of each OTU or genus in all subjects. RDA calculates p-values by permutating (Monte Carlo) the sample status.
qPCR gene copy counts of each target (total bacterial counts, EPEC *eaeA* gene, ETEC *lt* gene, ETEC *st* gene) were compared between test product dose groups by Kruskal-Wallis test with Dunn’s posthoc test and change over time were calculated by subtracting the counts at end-line from the counts at baseline. Pathogenic *E. coli* was defined as the sum of the gene copies of EPEC, ETEC lt and ETEC st).

*Table S1:* List of ingredients

| |
|---|
| Skimmed milk, Glucose syrup solids, Vegetable oils, Palm oil, Canola oil (low erucic acid type), Palm kernel oil, Sunflower oil, Saccharose, Fish oil, Sodium L-ascorbate, Emulsifier (Lecithin), Lactose, Taurine, Meso-inositol, Choline chloride, Ferrous sulphate, Vanilla flavour, DL M-tocopheryl acetate, Zinc sulphate, L-Ascorbyl palmitate, Nicotinamide, Manganese sulphate, Calcium D-pantothenate, Thiamin hydrochloride, Cupric sulphate, Retinyl-acetate, Pyridoxine hydrochloride, ß-carotene, Folic acid, Potassium iodide, Phytomenadione, D-Biotin, Cholecalciferol, Sodium selenite. |

***Table S2:** Baseline characteristics of the ITT and PP population*

| | ITT | PP |
|---|---|---|
| **n** | 165 | 105 |
| **Age (months)** | 20.15 ± 6.27 | 20.13 ± 6.24 |
| **Gender (boys/girls) (%)** | 44.8 / 55.2 | 45.7 / 54.3 |
| **Social class (upper/middle/lower) (%)** | 0.6 / 18.4 / 81.0 | 1.0 / 19.4 / 79.7 |
| **Religion (Muslim/Christian) (%)** | 71.0 / 29.0 | 68.9 / 31.1 |
| **Weight (kg)** | 8.9 ± 1.2 | 8.9 ± 1.2 |
| **Height (cm)** | 77.5 ± 4.9 | 77.6 ± 4.8 |
| **Weight for age Z- score** | -1.80 ± 0.56 | -1.78 ± 0.56 |
| **Height for age Z- score** | -1.80 ± 0.65 | -1.79 ± 0.59 |
| **Weight for height Z-score** | -1.24 ± 0.78 | -1.21 ± 0.74 |
| **Hb (g/dL)** | 10.1, 1.1 | 10.1, 1.3 |
| **Ferritin (μg/L)** | 38.6, 41.7 | 37.0, 42.6 |
| **CRP (mg/L)** | 1.7, 6.7 | 1.8, 6.4 |
| **Inflammation prevalence (n) (%)** | 30.6% (49) | 17.6% (18) |
| **Vitamin B12 deficiency (n) (%)** | 2.5% (3) | 2.7% (2) |
| **Folate deficiency (n) (%)** | 13.5% (14) | 12.1% (8) |

*Data are presented as median,IQR, percentages, or mean±SD.*

*Table S3*: Anaemia prevalence (%), Hb and ferritin concentrations (median, IQR) of the toddlers that were at baseline iron deficient or non-iron deficient, baseline and after 6 months of daily consumption of the multi-nutrient fortified dairy-based drink.

| | **Iron-deficient at baseline (n=12)** | | **Non-iron deficient at baseline (n=87)** | |
|---|---|---|---|---|
| | **pre** | **post** | **pre** | **post** |
| **Anemia prevalence (%)** | 100% (n=12) | 41.7% (n=5) | 100% (n=87) | 28.7% (n=25) |
| **Hb (g/dl)** | 10.2, 1.5 | 11.4, 1.6 | 10.2, 1.3 | 11.6, 1.4 |
| **Ferritin (μg/L)** | 14.0, 12.2 | 28.4, 7.5 | 42.6, 46.8 | 39.0, 32.5 |

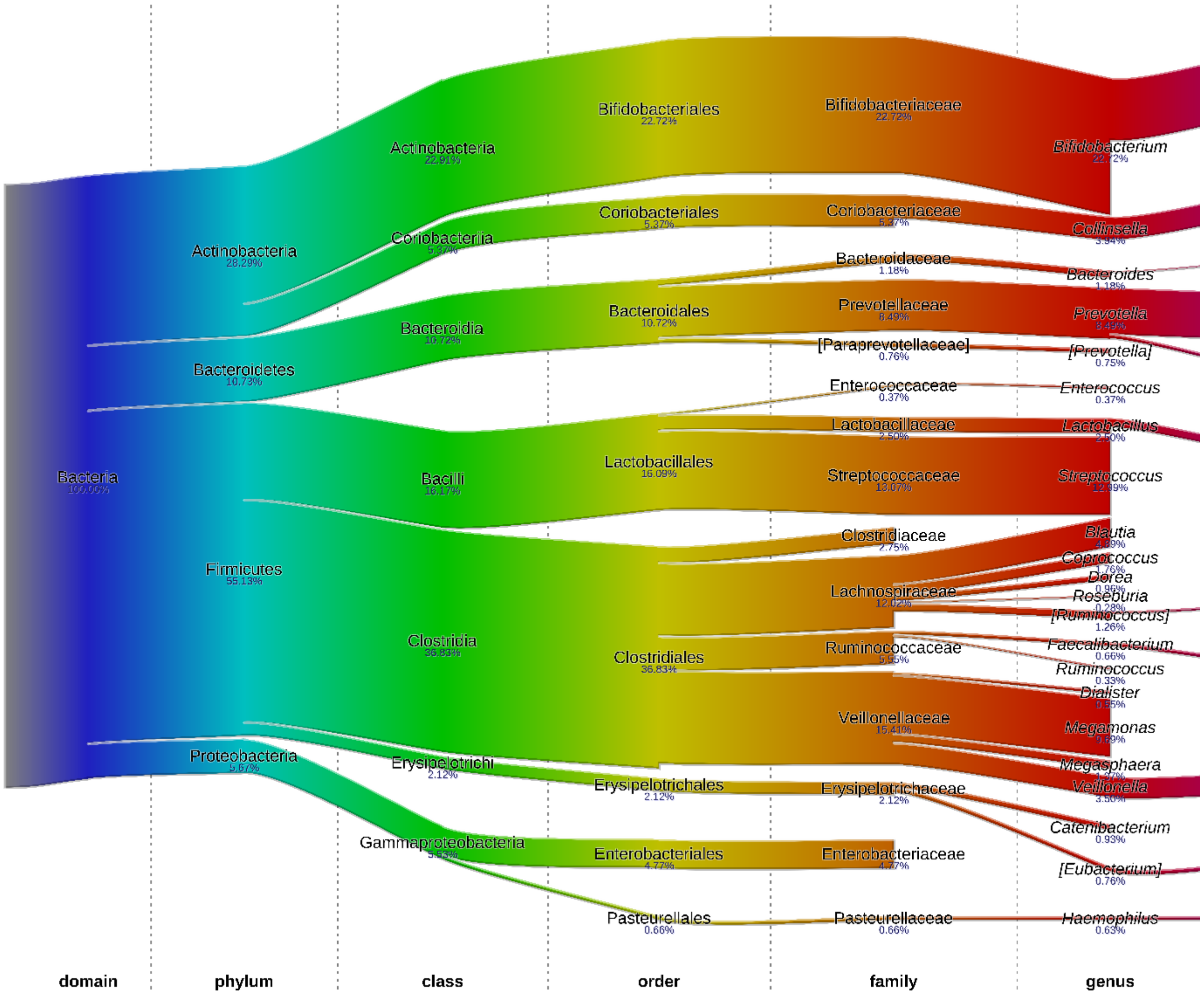


***Figure S1:*** Average gut microbiota composition of the studied toddler population at baseline, from the phylum to the genus level. The figure was generated using the software described by Sundquist *et al.*, 2007 [7].

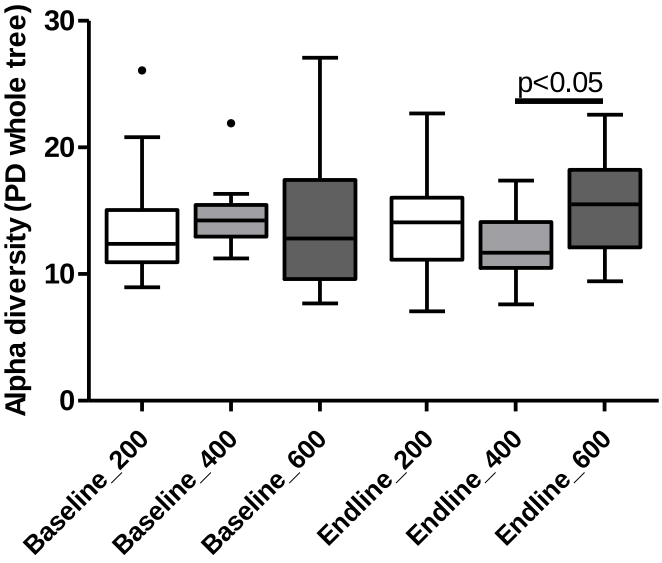


***Figure S2*****:** Alpha diversity (PD whole tree metric) in the multi-nutrient fortified dairy-based drink dose groups at baseline and endline. At endline the diversity was significantly lower in the 400 ml group compared to the 600 ml group (p<0.05 based on Dunn's posthoc test). Boxplots are displayed as Tukey whiskers.

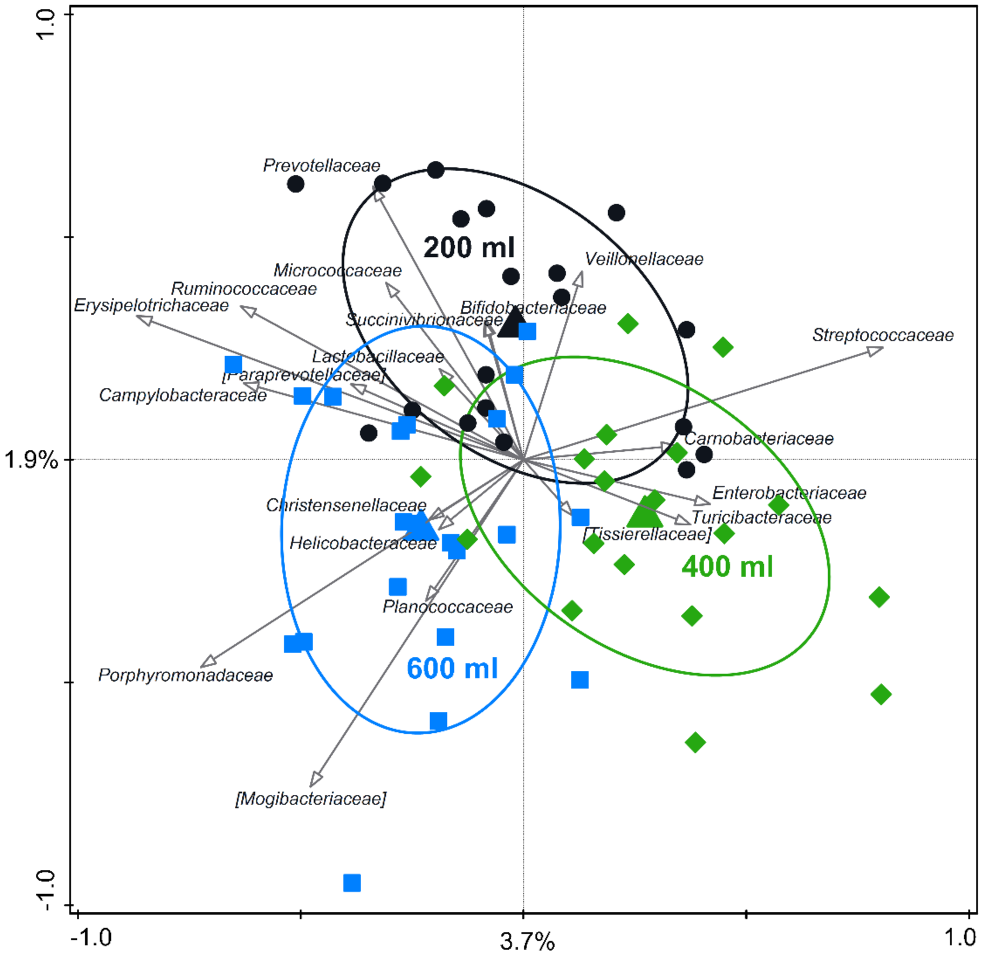


***Figure S3*:** Redundancy analysis (RDA) on the OTU level, assessing the effect of test product dose on gut microbiota composition at endline. OTUs were used as response data and test product dose was explanatory data, the bacterial families that contributed most were plotted supplementary. Variation explained by test product dose was 2.2%, p=0.006. Test product was a multi-nutrient fortified dairy-based drink

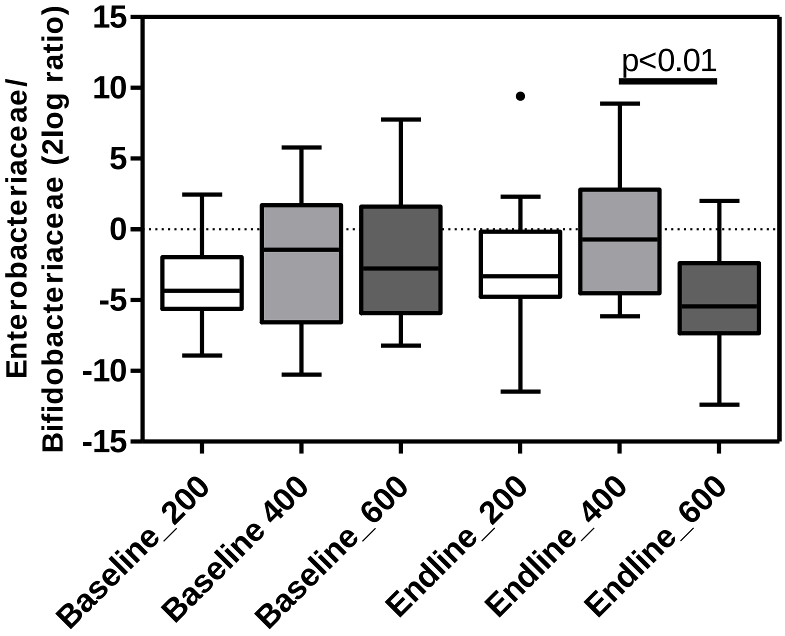


*Figure S4:*

*Enterobacteriaceae/Bifidobacteriaceae* ratio in the test product dose groups at baseline and endline. At endline the ratio was significantly higher in the 400 ml group compared to the 600 ml group ($p<0.01$ based on Dunn's posthoc test). Boxplots are displayed as Tukey whiskers. Test product was a multi-nutrient fortified dairy-based drink.

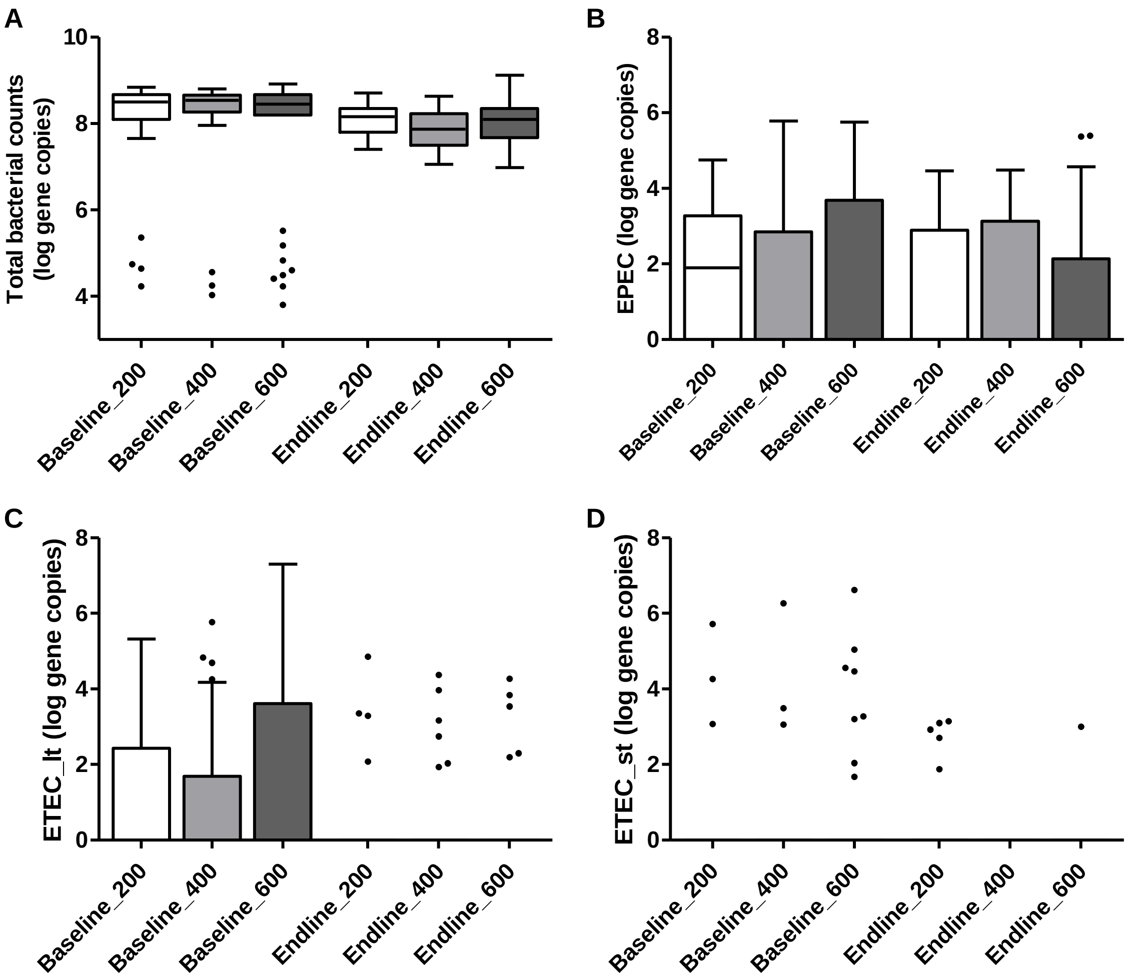


***Figure S5*:** Effect of daily intakes of 200 ml, 400 ml, or 600 ml of test product during 6 months on total bacterial counts (**A**) and pathogenic *E. coli*; EPEC (**B**), ETEC_lt (**C**), ETEC st (**D**) at baseline and endline. Test product was a multi-nutrient fortified dairy-based drink

Reference


1. Guion, C.E.; Ochoa, T.J.; Walker, C.M.; Barletta, F.; Cleary, T.G. Detection of diarrheagenic Escherichia coli by use of melting-curve analysis and real-time multiplex PCR. *J. Clin. Microbiol.* **2008**, *46*, 1752–1757, doi:10.1128/JCM.02341-07.

2. Suzuki, M.T.; Taylor, L.T.; DeLong, E.F. Quantitative analysis of small-subunit rRNA genes in mixed microbial populations via 5′-nuclease assays. *Appl. Environ. Microbiol.* **2000**, *66*, 4605–4614, doi:10.1128/AEM.66.11.4605-4614.2000.

3. Caporaso, J.G.; Kuczynski, J.; Stombaugh, J.; Bittinger, K.; Bushman, F.D.; Costello, E.K.; Fierer, N.; Pẽa, A.G.; Goodrich, J.K.; Gordon, J.I.; et al. QIIME allows analysis of high-throughput community sequencing data. *Nat. Methods* 2010, *7*, 335–336.

4. Edgar, R.C.; Haas, B.J.; Clemente, J.C.; Quince, C.; Knight, R. UCHIME improves sensitivity and speed of chimera detection. *Bioinformatics* **2011**, *27*, 2194–2200, doi:10.1093/bioinformatics/btr381.

5. Wang, Q.; Garrity, G.M.; Tiedje, J.M.; Cole, J.R. Naïve Bayesian classifier for rapid assignment of rRNA sequences into the new bacterial taxonomy. *Appl. Environ. Microbiol.* **2007**, *73*, 5261–5267, doi:10.1128/AEM.00062-07.

6. Braak, C.; Šmilauer, P. Canoco Reference Manual and User's Guide: Software for Ordination (version 5.0). *undefined* **2012**.

7. Sundquist, A.; Bigdeli, S.; Jalili, R.; Druzin, M.L.; Waller, S.; Pullen, K.M.; El-Sayed, Y.Y.; Taslimi, M.M.; Batzoglou, S.; Ronaghi, M. Bacterial flora-typing with targeted, chip-based Pyrosequencing. *BMC Microbiol.* **2007**, *7*, 108–108, doi:10.1186/1471-2180-7-108.
